# The value of hospital personnel serological screening in an integrated COVID-19 infection prevention and control strategy

**DOI:** 10.1017/ice.2020.242

**Published:** 2020-05-15

**Authors:** Filippo Quattrone, Marco Vabanesi, Alice Borghini, Giuseppe De Vito, Michele Emdin, Claudio Passino

**Affiliations:** 1 Institute of Life Sciences, Scuola Superiore Sant’Anna, Pisa, Italy; 2 Department of Translational Research and New Technologies in Medicine and Surgery, University of Pisa, Pisa, Italy; 3 Fondazione Toscana Gabriele Monasterio, Pisa, Italy; 4 Department of Neurology, San Raffaele Scientific Institute and University Hospital, Milan, Italy; 5 Institute of Management, Scuola Superiore Sant’Anna, Pisa, Italy; 6 Fondazione Stella Maris, Pisa, Italy

*To the Editor*—Severe acute respiratory syndrome coronavirus 2 (SARS-CoV-2) is highly infectious in healthcare-related settings, both among patients and healthcare workers (HCWs).^1^ Hospital personnel have shown an increased risk of coronavirus disease 2019 (COVID-19) compared to the general population, possibly associated with repeated exposures and, in the current emergency context, frequent lack of adequate personal protective equipment (PPE). The spread of SARS-CoV-2 has also been dramatically efficient in long-term care facilities (LTCFs), where the combination of asymptomatic or paucisymptomatic occupational carriers and a highly fragile elderly population have produced numerous outbreaks, greatly contributing to the total burden of COVID-19–related deaths.^2^

An integrated COVID-19 infection and prevention control (IPC) strategy must be promptly adopted by healthcare facilities to prevent further outbreaks. This strategy should involve HCWs, patients, visitors, and support personnel (eg, administrative and ancillary services workers) due to their reported role in nosocomial outbreaks.^3^ Current recommendations^4^ include the adoption of general IPC measures (eg, hand hygiene, physical distancing, universal use of surgical masks, and triage at entrance for fever, respiratory symptoms, and history of exposure to the virus), environmental measures (eg, enhanced surface cleaning, control of indoor air, proper linen, laundry, and waste management), administrative measures (eg, limiting visitor access and promoting remote work and telemedicine), and patient management measures (eg, dedicated pathways and isolation wards for patients with fever and respiratory symptoms, consistent use of adequate PPE, and universal SARS-CoV-2 screening for inpatients).

Systematic screening of HCWs and support personnel plays a key role in limiting the intrahospital spread of SARS-CoV-2. The most described approach is screening with viral genome real-time polymerase chain reaction (RT-PCR) on nasopharyngeal swabs. RT-PCR tests should be offered whenever an HCW presents with any symptom suggestive of COVID-19; initial screening is warranted for all new employees. Universal RT-PCR screening protocols^1,5^ have shown promising results. A limitation of this approach is the short-term RT-PCR positivity, with consequent need of repeated testing, sustained usage of intensive laboratory resources, exposure risks for the operators involved in screening, and possibly reduced compliance with repeated swab testing. To overcome some defects of traditional PCR-based testing, novel kits for point-of-care rapid PCR testing are currently being developed, with as yet uncertain yield.

Antibody response to SARS-CoV-2 has not been completely characterized; however, from the best available data,^6^ it appears that the detection of serum anti-SARS-CoV-2 IgG antibodies with appropriate methods (ie, chemiluminescence enzyme immunoassay, CLIA) is observable in almost all infected subjects within 20 days from symptom onset. Data on IgM appear less conclusive, and currently, these data do not support the classic sequential IgM–IgG transition; therefore IgM should not be the only target of antibody search.

Many regulatory institutes have assessed rapid, point-of-care antibody tests based on lateral flow immunoassay (LFIA), produced by multiple manufacturers. Although tempting for practical reasons, these tests have not met expectations for use in clinical settings due to unsatisfactory sensitivity and specificity.^7^

Serial serological screening with a validated technique, such as CLIA, could provide a significant contribution to IPC in hospitals and LTCFs, considering its lower cost, easier repeatability, and sustainability in the medium term, compared with swab-based molecular assays. Although serological tests have limited utility in diagnosing individual acute infections, they can inform actions to protect the hospital community. A serum antibody screening approach is indeed already used in surveillance campaigns among HCWs for other communicable diseases (eg, viral hepatitides).

**Fig. 1.**
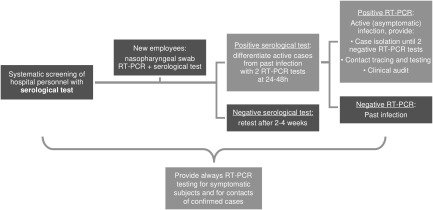
Hospital personnel serological screening in an integrated COVID-19 infection and prevention control strategy. Note: RT-PCR, real-time polymerase chain reaction.

A possible protocol (Fig. 1) could include systematic serological testing of all hospital personnel as well as subsequent second-line testing with viral RT-PCR to differentiate active cases from past infections. All IgG-negative subjects should be retested every 2–4 weeks according to the local epidemiological context and available resources. In case of seroconversion, an RT-PCR test is warranted. This approach would not substitute the standard, shorter-window RT-PCR testing of symptomatic subjects, but it would allow easier identification of asymptomatic carriers and guide subsequent contact tracing and testing, with more judicious resource usage compared to a hypothetical universal serial RT-PCR screening regimen.^8^

With the preliminary application of this protocol, starting in April 2020, we performed screening of ~800 HCWs in 3 non-COVID hospital centers in Tuscany, Italy. Thanks to serological screening and confirmatory RT-PCR swab tests at seroconversion, we detected 3 asymptomatic SARS-CoV-2 carriers with ongoing viral spread. Consequent epidemiological analyses documented probable nonoccupational origin of the 3 cases. In the context of an integrated IPC strategy, no secondary cases were detected among hospital strict contacts.

We believe that periodic serum antibody screening with validated techniques may be used to guide RT-PCR testing to detect asymptomatic or pauci-symptomatic infections and help stop nosocomial transmission chains. Further studies on effectiveness, acceptability, sustainability of this approach are warranted.
